# Tooth Wear Is Frequent in Adult Patients with Celiac Disease

**DOI:** 10.3390/nu9121321

**Published:** 2017-12-04

**Authors:** Massimo Amato, Fabiana Zingone, Mario Caggiano, Paola Iovino, Cristina Bucci, Carolina Ciacci

**Affiliations:** Department of Medicine, Surgery and Dentistry, Medical School of Salerno, 84131 Salerno, Italy; mamato@unisa.it (M.A.); fabiana.zingone@outlook.com (F.Z.); macaggiano@unisa.it (M.C.); piovino@unisa.it (P.I.); cristinabucci@hotmail.it (C.B.)

**Keywords:** enamel hypoplasia, tooth wear, aphthosis, celiac disease

## Abstract

(1) Background: Celiac disease (CD) patients can be affected by mouth and tooth disorders, which are influenced by their gluten-free diet. The aim of our research was to evaluate the pathological conditions of the stomatognathic system observed in celiac patients on a gluten-free diet. (2) Methods: we consecutively recruited celiac patients on a gluten-free diet at our celiac center, as well as healthy volunteers. Two dentists examined all patients/controls and checked them for any mouth disorder. (3) Results: Forty-nine patients affected by celiac disease (age at test 31.8 ± 11.58, time on GFD 8.73 ± 7.7) and 51 healthy volunteers (age at test 30.5 ± 8.7) were included. Recurrent aphthous stomatitis was reported in 26 patients (53.0%) and in 13 (25.5%) controls (*p* = 0.005). Dental enamel disorders were reported in 7 patients (14.3%) and in 0 controls (*p* = 0.002), with none having geographic tongue. We found non-specific tooth wear, characterized by loss of the mineralized tissue of the teeth, in 9 patients (18.3%) and in 3 (5.9%) controls (*p* = 0.05). (4) Conclusion: Recurrent aphthous stomatitis and enamel hypoplasia are “risk indicators” that may suggest that an individual has CD. We detected a high prevalence of non-specific tooth wear that can be caused by several factors such as malocclusion, sleep bruxism, parafunctional activity, and age.

## 1. Introduction

Celiac disease (CD) is an immune-mediated systemic disorder caused by gluten, a protein found in wheat and some related cereals. In genetically susceptible individuals, gluten induces CD-specific antibodies (anti-transglutaminase and anti-endomysial antibodies) and enteropathy [1,2].

The prevalence of CD is about 1% in Europe and the USA, and evidence shows that it is increasing worldwide [3,4,5]. The clinical presentation of CD ranges from a severe malabsorption syndrome to a number of extraintestinal symptoms [2]. Additionally, screening programs among first-degree relatives of patients with CD, or patients with other autoimmune disorders, aid CD diagnosis in subjects with subclinical presentation or no symptoms [6]. It is now widely recognized that mouth and tooth disorders can affect CD [7,8].

Enamel hypoplasia and recurrent aphthous stomatitis (RAS) have been the subject of many studies, with a particular focus on children. It is well-known that gluten exclusion does not influence the enamel hypoplasia, since the damage has already been established and it is irreversible [7,8], whereas RAS improves on a gluten-free diet (GFD) [9]. Atrophic glossitis (also known as geographical tongue) is instead reported in CD before diagnosis [10].

The aim of our study was to evaluate the pathological conditions of the stomatognathic system (teeth and oral mucosa) observed in CD patients on a GFD.

## 2. Materials and Methods

Over a period ranging from September 2015 to April 2016, we consecutively recruited a population composed of CD patients on a GFD, enrolled at the Gastroenterology Department of the University of Salerno, as well as healthy volunteers recruited among the hospital staff and the CD patients’ friends. Two dentists (MA and MC) examined all the patients together. They did not attend the gastroenterological visit, and therefore they did not know to which group the subjects belonged (patient or control).

All procedures performed in studies involving human participants were in accordance with the ethical standards of the institutional and/or national research committee and with the 1964 Helsinki declaration and its later amendments or comparable ethical standards. This study protocol was approved by Regione Campania, San Giovanni di Dio e Ruggi D’Aragona, Salerno Ethic Committee in date 29 October 2012 with approval code 836.

Written informed consent was obtained from all individual participants included in the study.

The inclusion criteria for the celiac group were: age older than 18 years; positive CD-specific serology (anti-transglutaminase IgA and antiendomysial IgA in absence of IgA deficiency) and positive intestinal biopsy, or a B-C grade histology according to Corazza-Villanacci classification [11] before starting the GFD. All CD patients had to have been on a GFD for at least one year and have been anti-transglutaminase IgA negative at the time of the examination. We excluded patients who had received the CD diagnosis before the age of 3 (when deciduous teeth should be completed).

Inclusion criteria for the control group were: age older than 18 years; negativity to anti-transglutaminase antibodies; no gastroenterological disease; no infectious, immune-mediated, or auto-immune disorder.

**Figure 1:**
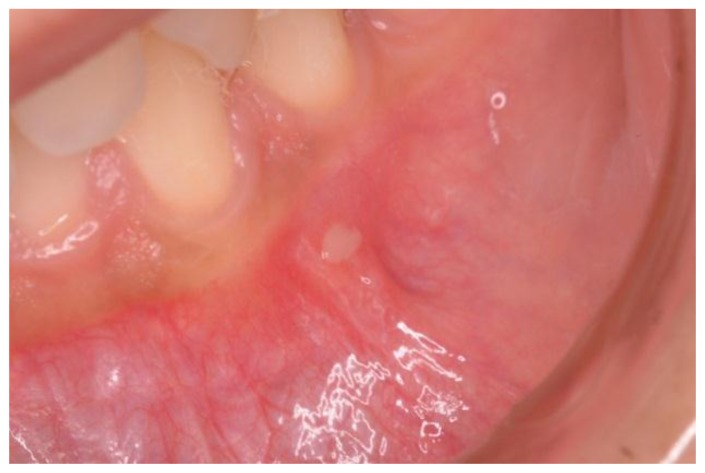
A case of aphthosis on the buccal mucosa.

Demographics and clinical information were collected during the gastroenterological visit, and data were recorded in a dedicated database. At the moment of the dental visit, patients were asked about recurrent aphthous stomatitis (RAS) characterized by recurrent bouts of solitary or multiple shallow painful ulcers, at intervals of a few months to a few days in patients who were otherwise well (Figure 1) [12]. Aphthosis at the time of the exam was also detected.

**Figure 2:**
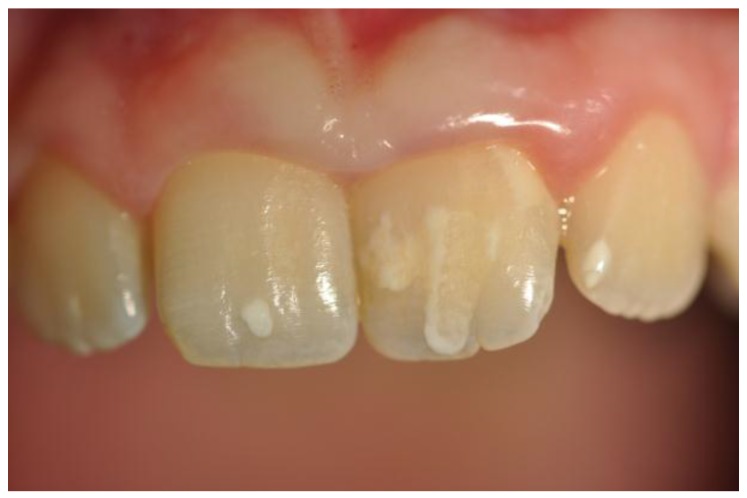
A case of enamel hypoplasia in a celiac disease (CD) patient.

The color, type, and site of dental enamel defects were recorded and classified according to Aine classification [8,13]. According to Aine, enamel defects appear symmetrically and chronologically in the same anatomical groups of teeth in all four quadrants of dentition, while single and asymmetrical changes were regarded as highly unspecific to CD. Enamel hypoplasia, in particular, is characterized by a reduced deposition of the enamel matrix by the ameloblasts. This alteration is clinically featured by depressions or grooves on the surface of the tooth (Figure 2).

Depending on the severity, it can vary from a mere change of color towards chalky white or gray and brownish, to areas of leakage of substances, up to severe cases of complete absence of enamel. Finally, the presence of atrophic glossitis (geographic tongue) was detected, which is characterized by glossodynia, atrophy of the filiform papillae, and erythematous areas on the back of the tongue.

### Statistical Analysis

Categorical and continuous variables were expressed as frequency and mean ± standard deviation (SD), respectively. Differences in frequencies between the groups were calculated using the *χ*^2^ test, while differences between continuous variables were calculated using the *t*-test. We compared the two groups according to age, sex, smoking habit, and alcohol consumption. All tests were two-tailed with significance level set at *p* < 0.05. All of the analyses were performed using Stata version 12, Stata Corp., College Station, TX, USA.

## 3. Results

Sixty-five CD patients consecutively attending the celiac center at the University of Salerno during the study period were eligible to participate in the study. However, thirteen patients refused to undergo the oral examination, and three had received the CD diagnosis before the age of three. Forty-nine people agreed to participate and were enrolled—37 females (75.5%) whose age at test was 31.8 ± 11.58 years, and whose time on GFD was 8.73 ± 7.7 years. Fifty-one controls were included in the study—31 females (60.8%), whose age at test was 30.5 ± 8.7 years. There were no statistically significant differences between the two groups in terms of sex, age, smoking habit, or alcohol consumption (*p* > 0.05). RAS was reported in 26 CD patients (53.0%) and in 13 (25.5%) controls (*p* = 0.005) (see Table 1).

Twenty-four CD patients reported recurrence of aphthosis before the CD diagnosis, while only two patients had had two aphthosis episodes within one year of diagnosis. No patient or control had aphthosis at the time of the dental examination. Dental enamel disorders were reported in seven celiac patients (14.3%) and in 0 controls (*p* = 0.002). In particular, four patients had a grade 1, according to Aine classification (defects in color of enamel, single or multiple cream, yellow, or brown opacities with clearly defined or diffuse margins), and three had a grade 2 according to Aine (slight structural defects, rough enamel surface filled with horizontal grooves or shallow pits) (see Table 1). As enamel hypoplasia is a sign of early gluten damage (before teething), we compared the age at diagnosis between those with and without enamel hypoplasia, but we did not observe any significant statistical difference between them (25.6 ± 10.6 vs. 22.48 ± 13.4, *p* = 0.5). None had atrophic glossitis at the time of the oral examination. During the dental examination, we accidentally found that nine CD patients (18.3%) and three (5.9%) controls (*p* = 0.05) showed non-specific tooth wear (see Table 1), characterized by loss of mineralized tooth tissue, unrelated to bacterial demineralization action. We adopted the Smith and Knight tooth wear index to classify these lesions [14].

**Figure 3:**
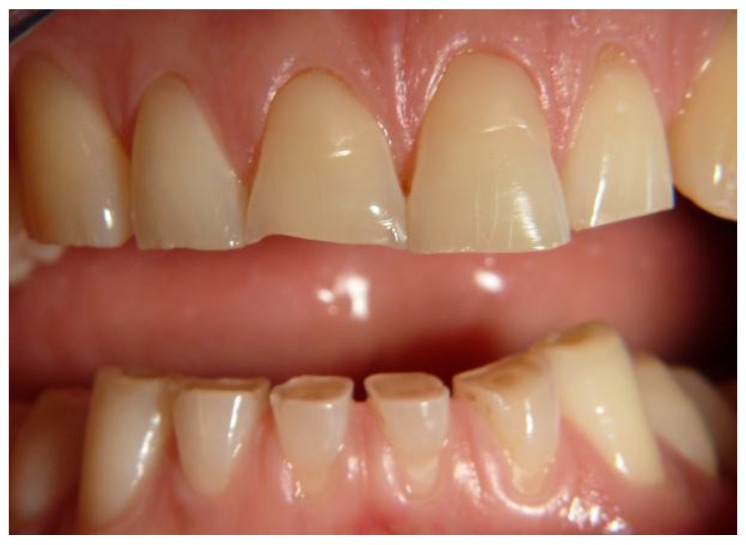
A case of tooth wear in a celiac disease (CD) patient (Score 3).

Following this classification we observed: Score 1 (loss of enamel characteristics) in four CD patients, Score 2 (exposing dentine for less than one-third of surface) in three, Score 3 (exposing dentine for more than one-third of surface) in two (Figure 3). In the control group, we only found three patients with tooth wear, all classified as Score 1.

## 4. Discussion

This case–control analysis confirmed the higher prevalence of enamel hypoplasia and RAS in CD patients compared to the general population, and evidenced the presence of a new possible sign of CD diagnosis based on the presence of non-specific tooth wear that was accidentally found during the dental visit. The strength of this study is that the two dentists—blinded to the subjects’ status—conducted the oral examination employing standardized methods. We exclusively included adult patients on a GFD to detect which disorders are unrelated to the elimination of gluten. However, the study can be considered limited due to the small size of the sample, which may have influenced our results, and because we did not have data on the dental disorders at the time of diagnosis.

Oral disorders have been associated with CD and other immune-mediated diseases, such as Crohn's disease [15,16]. Moreover, stress, anxiety, and sleep disturbances can also function as additional triggers [17]. In studies conducted on children and/or adults, CD and both enamel hypoplasia and RAS have been clearly associated. Table 2 and Table 3 summarize these studies published since the year 2000. Compared to these studies, we found a higher prevalence of RAS (53%) but a lower prevalence of enamel hypoplasia (14.3%).

In 1968, Lehner [18] first suggested that the aphthous lesions in undiagnosed CD patients could be the consequence of immunological mechanisms. In 1992, Majorana found the presence of aphthae in 19 (16.8%) out of 113 CD patients at diagnosis, and reported that the frequency of these lesions decreased while on a GFD [19]. Moreover, the study showed that the re-exposition to gluten determined the recurrence of the sores, as well as an increase in the level of antibodies and intestinal damage.

As shown in Table 2, an Italian case–control study carried out in 2007 showed that in children with CD, RAS had a higher frequency and a higher rate of recurrence compared to the control group [20]. Another Italian study further confirmed this finding, reporting that RAS was present in 21% (19/90) of CD adult patients and 0.5% (1/180) of controls [9]. A high frequency of RAS in CD was also reported by studies conducted in the USA (42.4% in CD vs. 23.2% in controls) [21], Brazil (40.3% in celiac and 17.3% in control) [22], and Turkey (44% in celiac and 0% in control) [23].

Contrary to these data, a previous study failed to demonstrate the association between CD and RAS (33.3% of celiac patients and 23.4% of controls, *p* > 0.05) [24].

Enamel hypoplasia in the permanent dentition is usually bilateral, symmetrical, more frequently found on the central incisors, upper lateral, and lower ones, the cusps of the first molars and canines, and pre-molars. To-date, the pathogenesis of enamel defects in CD has not had a clear explanation. The more accredited hypothesis is that the damage is the effect of poor mineralization caused by the malabsorption of calcium, phosphate, and vitamin D [24,27,28]. The low concentration of calcium caused by intestinal malabsorption and observed in patients during tooth development is indeed involved in the development of enamel hypoplasia [28,29].

However, different immune or genetic genesis of lesions cannot be excluded. T-lymphocytes may have a role in attacking the enamel [30,31], and there may be an association between dental enamel effects and the haplotype HLA-DR3 even in the general population. On the contrary, the DR5-DR7 phenotype has been associated with protection from damage to the enamel, explaining why not all CD patients suffer from enamel hypoplasia [19,30].

Other systemic factors (e.g., malnutrition and reduced concentration of vitamin D and A) are possibly involved in enamel hypoplasia in CD and other diseases causing malnutrition. The prevalence of dental enamel hypoplasia/defects varies among the studies.

Aguirre et al. noted that enamel defects were observed in 72 (52.5%) patients with CD and 22 (42.3%) control subjects (*p* = 0.006); they mostly affected the incisors (70.8%) and canines (20.8%) at the level of a permanent incisal third of the crown [32]. Italian studies based on children and/or adults found a prevalence of enamel hypoplasia ranging between 20% and 85.2% in CD patients [8,9,20,25,26]. Other studies confirmed that defects in the development of the enamel are more frequently found in patients with CD rather than in healthy individuals [21,23,33,34] (see Table 3).

It has been suggested that a higher prevalence of enamel lesions might be more common in patients with non-gastrointestinal complaints of CD, compared to those expressing the typical malabsorption syndrome [35]. A structural difference between the enamel hypoplasia observed in CD patients and other individuals has been reported, showing that CD patients have a reduced degree of mineralization of the enamel, shorter prisms, and a lower content of interprismatic substance, which is even more asymmetrically distributed than in non-CD subjects [35].

The new finding of our study is that we detected a high prevalence of non-specific tooth wear which can be caused by several factors, including malocclusion, sleep bruxism, parafunctional activity, and age [36]. The tooth wear has been associated with sleep disorders, eating disorders, and psychological problems [37,38,39]; similarly, CD is a condition that is associated with psychological impairment in both untreated and treated conditions [40,41,42,43]. Future prospective studies should therefore analyze this possible association in CD.

In conclusion, RAS and enamel hypoplasia have been found more in CD patients than in controls, and they can be considered as “risk indicators”; these may suggest that an individual has CD, and thus prompt a focused work-up. Moreover, future studies should analyze the significance of non-specific tooth wear in CD patients and their association with psychological disorders in patients with CD pre- and post-diagnosis.

## Figures and Tables

**Table 1:** Lesions found during the dental examination in cases and controls.

	CD Patients (49) *n* (%)	Controls (51) *n* (%)	*p*
RAS	26 (53)	13 (25.5)	0.005
Aphthosis during visit	0	0	
Enamel hypoplasia	7 (14.3)	0	0.002
Aine grade 1	4		
Aine grade 2	3		
Aine grade 3	0		
Aine grade 4	0		
Atrophic glossitis	0	0	
Non-specific enamel wear	9 (18.3)	3 (5.9)	0.05
Smith and Knight index grade 1	4	3	
Smith and Knight index grade 2	3	0	
Smith and Knight index grade 3	2	0	
Smith and Knight index grade 4	0	0	

CD, celiac disease; RAS, recurrent aphthous stomatitis.

**Table 2:** Previous literature on the association between celiac disease (CD) and recurrent aphthous stomatitis (RAS).

Author	Country	Number of Subjects	Population studied	Prevalence of RAS Detected
Bucci 2006 [24]	Italy	CD Patients: 72 Controls: 162	Children	CD Patients: 33.3% Controls: 23.4%
Campisi 2007 [9]	Italy	CD Patients: (90/107) Controls: (180/233)	Adults and Children	Adult CD Patients: 21% Adult Controls: 0.5% Pediatric CD Patients: 17% Pediatric Controls: 1%
Procaccini 2007 [20]	Italy	CD Patients: 50 Controls: 50	Children/young adult	CD Patients: 36% Controls: 12%
Cheng 2010 [21]	USA	CD Patients: 67 Controls: 69	Adults and Children	CD Patients: 42.4% Controls: 23.2%
Costacurta 2010 [25]	Italy	CD Patients: 300 Controls: 300	Children	CD Patients: 8.3% Controls: 3%
Erriu 2013 [26]	Italy	CD Patients: 44	Children	CD Patients: 18.2%
de Carvalho 2015 [22]	Brazil	CD Patients: 52 Controls: 52	Children	CD Patients: 40.3% Controls: 17.3%
Cantekin 2015 [23]	Turkey	CD Patients: 25 Controls: 25	Children	CD Patients: 44% Controls: 0%
This Study 2017	Italy	CD Patients: 49 Controls: 51	Adults	CD Patients: 53% Controls: 25.5%

GFD: gluten-free diet.

**Table 3:** Previous literature on the association between celiac disease (CD) and enamel hypoplasia.

Author	Country	Number of Subjects	Population studied	Prevalence of Enamel Defects
Bucci 2006 [24]	Italy	CD Patients: 72 Controls: 162	Children	CD Patients: 20% Controls: 5.6%
Campisi 2007 [9]	Italy	CD Patients: (90/107) Controls: (180/233)	Adults and Children	Adult CD Patients: 23% Adult Controls: 8% Pediatric CD Patients: 23% Pediatric Controls: 9%
Wierink 2007 [34]	Netherlands	CD Patients: 53 Controls: 28	Children	CD Patients: 55% Controls: 18%
Procaccini 2007 [20]	Italy	CD Patients: 50 Controls: 50	Children/young adult	CD Patients: 26% Controls: 16%
Ortega Paez 2008 [33]	Spain	CD Patients: 30 Controls: 30	Children	CD Patients: 83.3% Controls: 53.3%
Cheng 2010 [21]	USA	CD Patients: 44/23 Controls: 45/24	Adults and Children	Adult CD Patients:32% Adult Controls: 29% Pediatric CD Patients: 87% Pediatric Controls: 33%
Costacurta 2010 [25]	Italy	CD Patients: 300 Controls: 300	Children	CD Patients: 33% Controls: 11%
Erriu 2013 [26]	Italy	CD Patients: 44	Children	CD Patients: 38.6%
Trotta 2014 [8]	Italy	CD Patients: 54	Adults	CD Patients: 85.2%
de Carvalho 2015 [22]	Brazil	CD Patients: 52 Controls: 52	Children	CD Patients: 61.5% Controls: 21.1%
Cantekin 2015 [23]	Turkey	CD Patients: 25 Controls: 25	Children	CD Patients: 48% Controls: 16%
This Study 2017	Italy	CD Patients: 49 Controls: 51	Adults	CD Patients: 14.3% Controls: 0%

GFD: gluten-free diet.
